# Supplementary material for: Stimulator of Interferon Genes Protein (STING) Expression in Cancer Cells: A Tissue Microarray Study Evaluating More than 18,000 Tumors from 139 Different Tumor Entities
Source: Cancers (Basel). 2024 Jun 30;16(13):2425. doi: 10.3390/cancers16132425 (PMC11240524; doi:10.3390/cancers16132425)
Supplement: Supplementary file 1 [file cancers-16-02425-s001.zip › cancers-3055431-supplementary/Suppl table S1_STING_R1.pdf]

**Staining intensity**	**Fraction of stained tumor cells**	**Score**
0	0	negative
1+	≤70%	weak
2+	≤30%	
1+	>70%	moderate
2+	>30 but ≥70%	
3+	≤30%	
2+	>70%	strong
3+	>30%	
