# Supplementary material for: Stimulator of Interferon Genes Protein (STING) Expression in Cancer Cells: A Tissue Microarray Study Evaluating More than 18,000 Tumors from 139 Different Tumor Entities
Source: Cancers (Basel). 2024 Jun 30;16(13):2425. doi: 10.3390/cancers16132425 (PMC11240524; doi:10.3390/cancers16132425)
Supplement: Supplementary file 1 [file cancers-16-02425-s001.zip › cancers-3055431-supplementary/Suppl table S2_STING.pdf]

Tumor entity	HPV status	STING immunostaining					
		n	negative (%)	weak (%)	moderate (%)	strong (%)	P
All squamous cell cancers	negative	263	17.5	43.3	20.5	18.6	0.0212
	positive	217	12.4	35.5	23.0	29.0	
Oral squamous cell carcinoma	negative	57	17.5	36.8	21.1	24.6	0.7699
	positive	10	10.0	30.0	20.0	40.0	
Squamous cell carcinoma of the pharynx	negative	19	0.0	63.2	21.1	15.8	0.0390
	positive	31	3.2	25.8	25.8	45.2	
Squamous cell carcinoma of the larynx	negative	45	17.8	37.8	17.8	26.7	0.6730
	positive	8	37.5	25.0	12.5	25.0	
Squamous cell carcinoma of the cervix	negative	8	0.0	37.5	37.5	25.0	0.5432
	positive	67	11.9	32.8	26.9	28.4	
Squamous cell carcinoma of the vagina	negative	15	6.7	40.0	20.0	33.3	0.1822
	positive	14	14.3	57.1	0.0	28.6	
Squamous cell carcinoma of the vulva	negative	54	22.2	44.4	20.4	13.0	0.7033
	positive	24	33.3	41.7	12.5	12.5	
Squamous cell carcinoma of the penis	negative	27	11.1	48.1	29.6	11.1	0.4949
	positive	34	8.8	38.2	26.5	26.5	
Squamous cell carcinoma of the skin	negative	34	32.4	47.1	11.8	8.8	0.2532
	positive	1	0.0	0.0	100.0	0.0	
Squamous cell carcinoma of the anal canal	negative	4	25.0	50.0	25.0	0.0	0.2725
	positive	28	3.6	39.3	28.6	28.6	
