# Supplementary figures and images for: Stimulator of Interferon Genes Protein (STING) Expression in Cancer Cells: A Tissue Microarray Study Evaluating More than 18,000 Tumors from 139 Different Tumor Entities
Source: Cancers (Basel). 2024 Jun 30;16(13):2425. doi: 10.3390/cancers16132425 (PMC11240524; doi:10.3390/cancers16132425)

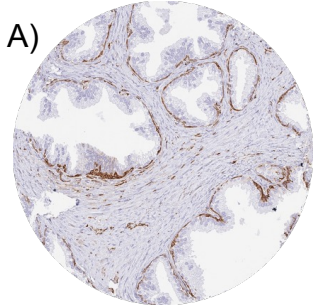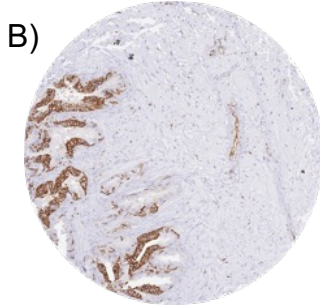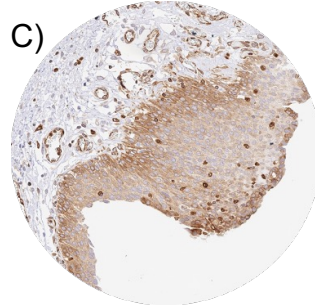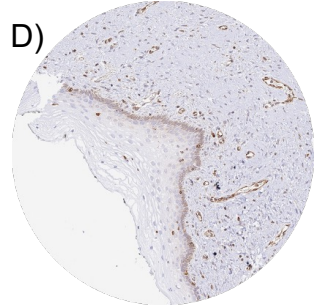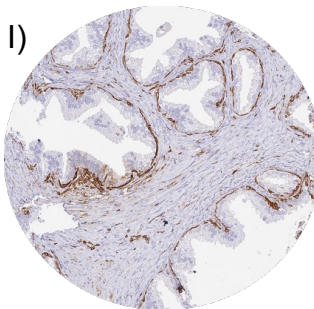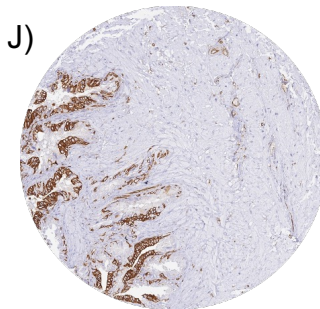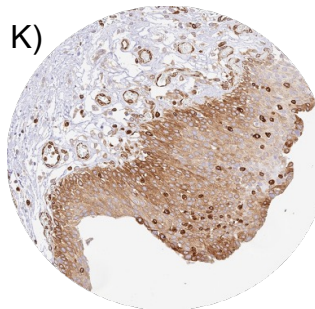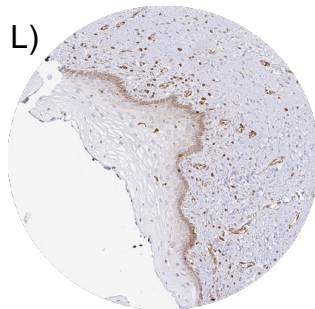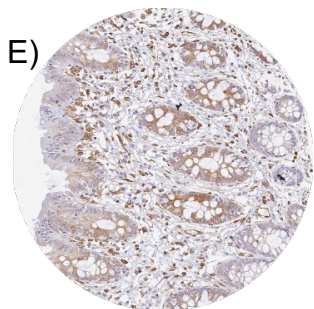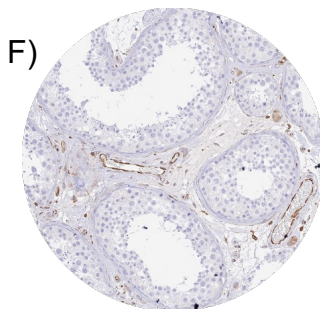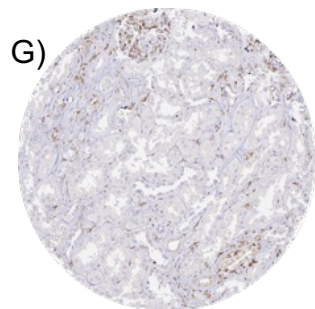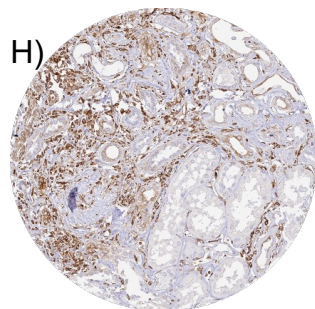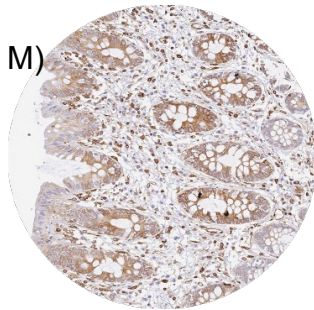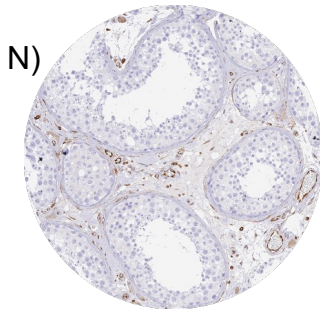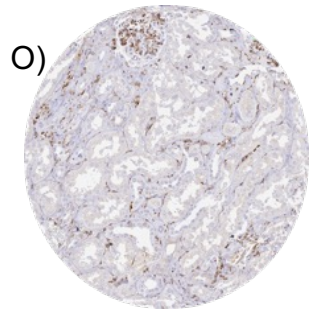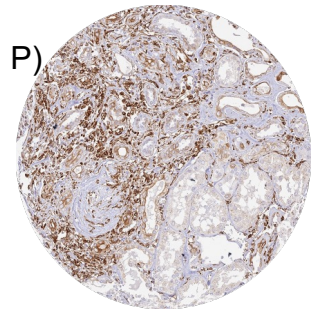

Supplement: Supplementary file 1 [file cancers-16-02425-s001.zip › cancers-3055431-supplementary/Suppl Figure S1_STING.pdf]

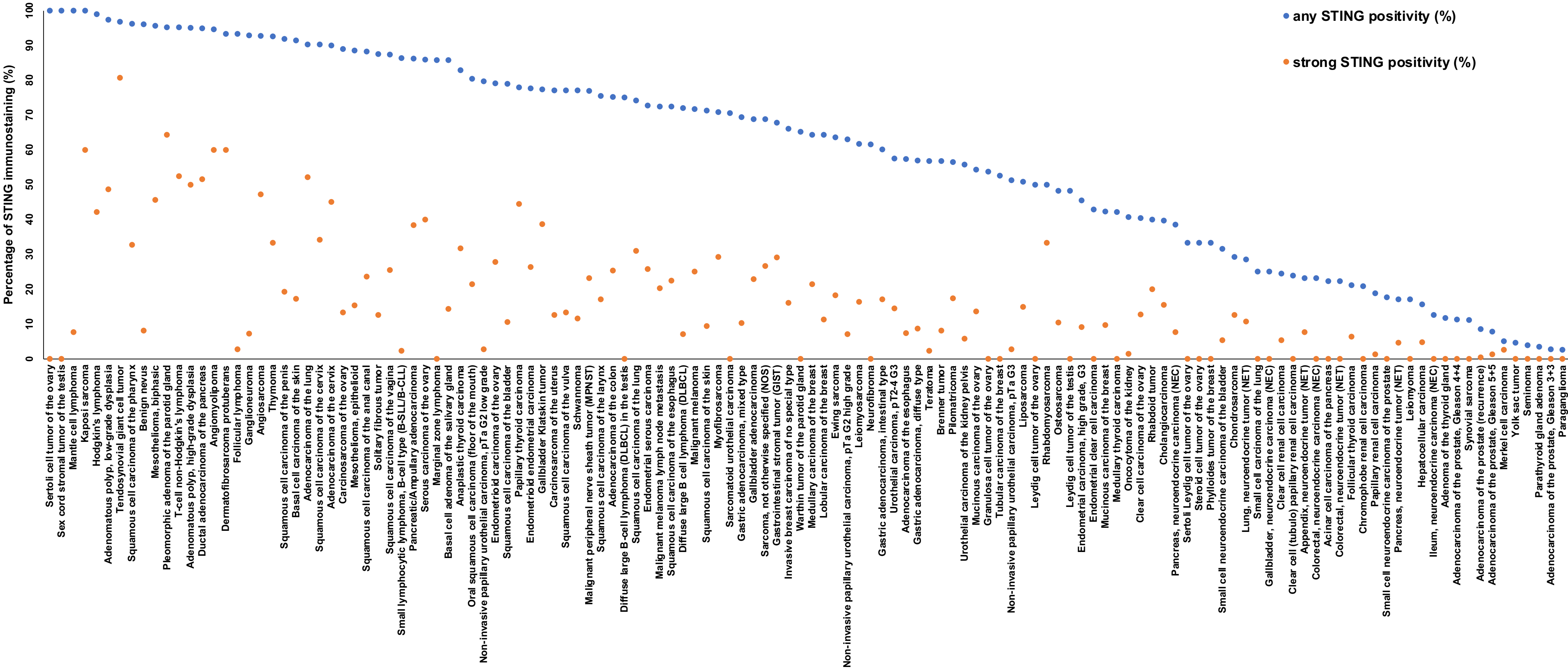

Supplement: Supplementary file 1 [file cancers-16-02425-s001.zip › cancers-3055431-supplementary/Suppl Figure S2_STING.pdf]

a)

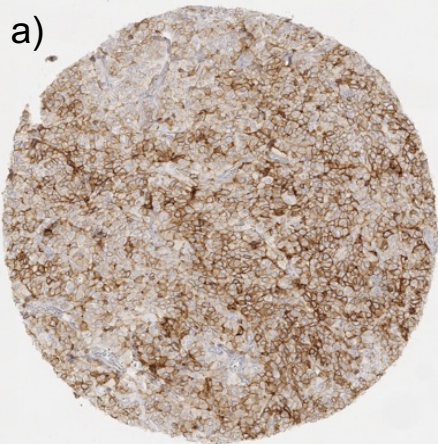

b)

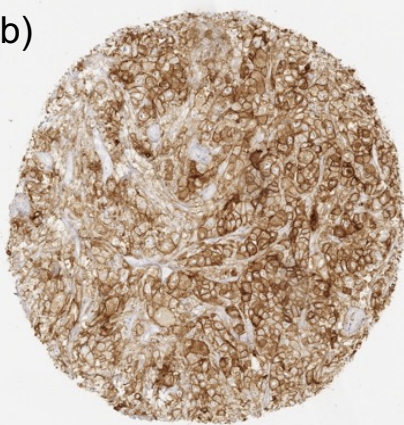

c)

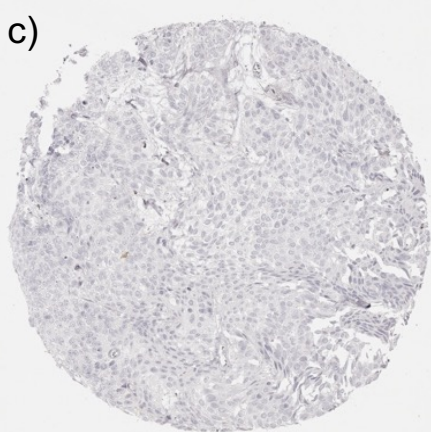

d)

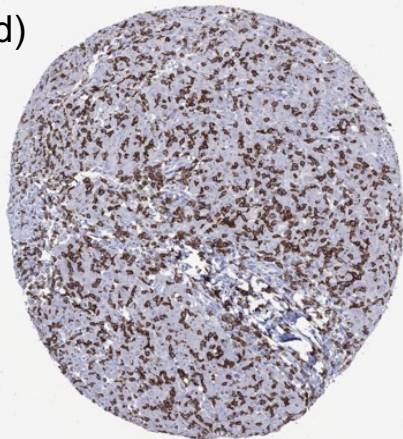

e)

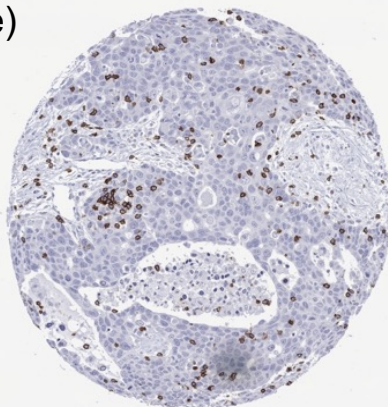

f)

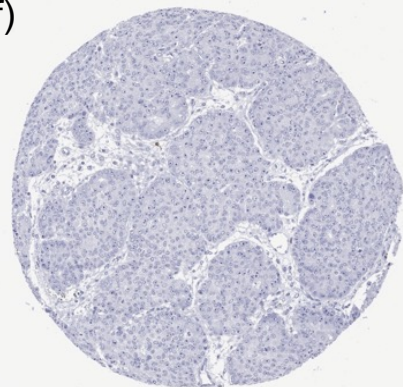

Supplement: Supplementary file 1 [file cancers-16-02425-s001.zip › cancers-3055431-supplementary/Suppl Figure S3_STING_R1.pdf]
