# Supplementary material for: Stimulator of Interferon Genes Protein (STING) Expression in Cancer Cells: A Tissue Microarray Study Evaluating More than 18,000 Tumors from 139 Different Tumor Entities
Source: Cancers (Basel). 2024 Jun 30;16(13):2425. doi: 10.3390/cancers16132425 (PMC11240524; doi:10.3390/cancers16132425)
Supplement: Supplementary file 1 [file Suppl table S3_STING.pdf › cancers-3055431-supplementary/Suppl table S3_STING.pdf]

Tumor entity	Pathological and molecular parameters	n	STING immunostaining negative (%)	weak (%)	moderate (%)	strong (%)	P
Papillary renal cell carcinoma	ISUP 1	39	87.2	12.8	0.0	0.0	0.5368
	ISUP 2	127	85.0	10.2	3.9	0.8	
	ISUP 3	72	76.4	15.3	6.9	1.4	
	ISUP 4	7	71.4	14.3	14.3	0.0	
	Fuhrman 1	2	50.0	0.0	0.0	50.0	0.1195
	Fuhrman 2	178	83.1	13.5	2.8	0.6	
	Fuhrman 3	72	80.6	12.5	5.6	1.4	
	Fuhrman 4	11	63.6	9.1	18.2	9.1	
	Thoenes 1	55	81.8	14.5	1.8	1.8	0.1699
	Thoenes 2	146	82.9	13.0	3.4	0.7	
	Thoenes 3	16	56.3	25.0	6.3	12.5	
	UICC 1	96	85.4	9.4	4.2	1.0	0.6264
	UICC 2	14	78.6	21.4	0.0	0.0	
	UICC 3	5	100.0	0.0	0.0	0.0	
	UICC 4	12	66.7	25.0	8.3	0.0	
	pT1	193	82.4	13.5	3.1	1.0	0.6896
	pT2	45	80.0	15.6	2.2	2.2	
	pT3-4	33	78.8	9.1	9.1	3.0	
	pN0	24	87.5	12.5	0.0	0.0	0.0353
	pN+	15	60.0	13.3	20.0	6.7	
	pM0	27	85.2	14.8	0.0	0.0	0.0641
	pM+	11	63.6	18.2	18.2	0.0	
Adenocarcinoma of the pancreas	pT1	13	0.0	0.0	38.5	61.5	0.2376
	pT2	67	1.5	20.9	28.4	49.3	
	pT3	369	6.0	15.4	27.4	51.2	
	pT4	27	7.4	22.2	29.6	40.7	
	G1	16	6.3	37.5	37.5	18.8	0.2116
	G2	338	5.3	16.0	27.2	51.5	
	G3	100	5.0	16.0	31.0	48.0	
	pN0	105	9.5	15.2	23.8	51.4	0.1777
	pN+	370	4.1	16.5	28.9	50.5	
	R0	239	5.4	16.7	26.4	51.5	0.8482
	R1	199	5.0	17.6	29.6	47.7	
	MMR proficient	409	4.4	16.1	29.3	50.1	0.1200
	MMR deficient	4	25.0	0.0	0.0	75.0	
Adenocarcinoma of the stomach	pT1-2	44	29.5	45.5	11.4	13.6	0.7133
	pT3	109	27.5	36.7	16.5	19.3	
	pT4	92	33.7	38.0	16.3	12.0	
	pN0	60	28.3	40.0	16.7	15.0	0.9826
	pN+	184	30.4	38.6	15.2	15.8	
	MMR proficient	207	34.3	35.7	15.0	15.0	
	MMR deficient	36	27.8	33.3	11.1	27.8	0.3365
Endometrioid endometrial carcinoma	pT1	87	23.0	27.6	23.0	26.4	0.1585
	pT2	21	9.5	28.6	38.1	23.8	
	pT3-4	28	32.1	28.6	7.1	32.1	
	pN0	39	30.8	23.1	20.5	25.6	0.7539
	pN+	24	20.8	29.2	16.7	33.3	
Endometrioid carcinoma of the ovary	pT1	24	20.8	20.8	20.8	37.5	0.8378
	pT2	4	0.0	25.0	25.0	50.0	
	pT3	5	20.0	20.0	40.0	20.0	
	pN0	20	15.0	15.0	25.0	45.0	0.4544
	pN1	8	25.0	37.5	12.5	25.0	
Serous carcinoma of the ovary	pT1	35	14.3	22.9	5.7	57.1	0.1219
	pT2	41	19.5	E764.4	12.2	43.9	
	pT3	253	11.9	30.0	19.8	38.3	
	pN0	80	8.8	36.3	18.8	36.3	0.2592
	pN1	159	17.0	27.7	18.2	37.1	

abbreviation: pT: pathological tumor stage, G: Grade, pN: pathological lymph node status, pM: pathological status of distant metastasis, R: resection margin status, MMR: mismatch repair, ISUP: International Socity of Urological Pathology, UICC: Union for International Cancer Control
